# Functional Links between Biomass Production and Decomposition of Vetiver (*Chrysopogon zizanioides*) Grass in Three Australian Soils

**DOI:** 10.3390/plants11060778

**Published:** 2022-03-15

**Authors:** Bezaye Tessema, Brian Wilson, Heiko Daniel, Paul Kristiansen, Jeff A. Baldock

**Affiliations:** 1 School of Environmental and Rural Science, University of New England, Armidale, NSW 2351, Australia; bwilson7@une.edu.au (B.W.); hdaniel@une.edu.au (H.D.); pkristi2@une.edu.au (P.K.); 2 Soil and Water Research, Ethiopian Institute of Agricultural Research, Addis Ababa P.O. Box 2003, Ethiopia; 3 ARUA Water CoE, Institute for Water Research, Rhodes University, Grahamstown 6140, South Africa; 4 NSW Office of Environment and Heritage, Armidale, NSW 2351, Australia; 5 CSIRO Agriculture, Glen Osmond, SA 5064, Australia; jeff.baldock@csiro.au

**Keywords:** soil carbon, root, shoot, soil type, sand, silt, clay, Gunnedah

## Abstract

Plant roots are primary factors to contribute to surface and deep soil carbon sequestration (SCS). Perennial grasses like vetiver produce large and deep root system and are likely to contribute significantly to soil carbon. However, we have limited knowledge on how root and shoot decomposition differ and their contribution to SCS. This study examined biomass production and relative decomposition of vetiver which was grown under glasshouse conditions. Subsequently the biomass incubated for 206 days, and the gas analysed using ANCA-GSL. The results confirmed large shoot and root production potential of 161 and 107 Mg ha^−1^ (fresh) and 67.7 and 52.5 Mg ha^−1^ (dry) biomass, respectively with 1:1.43 (fresh) and 1:1.25 (dry) production ratio. Vetiver roots decomposed more rapidly in the clay soil (*p* < 0.001) compared with the shoots, which could be attributed to the lower C:N ratio of roots than the shoots. The large root biomass produced does indeed contribute more to the soil carbon accumulation and the faster root decomposition is crucial in releasing the carbon in the root exudates and would also speed up its contribution to stable SOM. Hence, planting vetiver and similar tropical perennial grasses on degraded and less fertile soils could be a good strategy to rehabilitate degraded soils and for SCS.

## 1. Introduction

Soils globally are important in sequestering atmospheric carbon and can thus significantly affect greenhouse gas flux [1,2,3]. Retention of organic matter (OM) in soil is however, largely controlled by environmental variables, the nature of the OM, and its spatial distribution and interactions with other soil constituents. Therefore, maximizing the carbon input, and minimizing the rate of organic matter decomposition after deposition in soil, are two important factors that can help to increase the amount of carbon sequestered from the atmosphere [4].

Plant production and decomposition determine carbon inputs to the soil profile, and as such, plant shoot and root allocation (above- and below-ground, respectively), as well as allocation of roots between shallow and deep soil layers, can result in a very different soil carbon distribution with depth in the profile [5]. Tropical perennial grasses grow continually and are adapted to a wide range of soil and climate conditions [4,6]. For many years, the International Center for Tropical Agriculture (CIAT) has been working on selecting tropical grasses with deep and massive root systems that can exploit nutrients and water from deeper soil profiles [7,8]. McKenzie and Mason [6], indicated that deep soil profiles with fertile subsoil allow deep root penetration into the subsoil where the environment is cooler and less likely to promote organic carbon decomposition than in topsoils. Belowground biomass is therefore believed to be a primary vehicle for soil carbon storage [9,10,11]. Hence, perennial grasses, due to their deep root systems, might contribute significantly to soil carbon [7,8], via biomass inputs and slow mineralization processes due to slow OM turnover at depth [12]. Studies also report that a large root biomass can support substantial soil microorganism populations and their metabolic processes, and thus contribute significantly to soil organic matter decomposition and carbon turnover [9]. A precise relationship between root biomass and soil organic carbon [13] is not, however, easy to establish because soil OM decomposition depends on several interacting factors including climate, litter quality, water and nutrient availability, soil type/texture and biotic activity [14,15,16].

Litter quality factors important to decomposition and mineralization include the chemical composition of the organic matter (e.g., C:N ratio), whereby litter with higher concentrations of nutrients and lower concentrations of lignin will decompose more rapidly [17]. Soil texture, and in particular clay content, can assist in the physical protection of SOC within soil aggregates and therefore suppress decomposition and promote SOC storage [18]. Bacteria and fungi are primary decomposers in soils, and soil structure and texture can be a dominant control over decomposition as they affect accessibility of microbes to the soil substrate and OM. These factors are reflected in different decomposition rates between different types of soil [19]. However, in decomposition studies, much attention has been given to biotic and abiotic (temperature and moisture) factors rather than soil structure and texture which are clearly linked [19,20]. Clay content is associated with factors such as plant growth and moisture and a larger retention of carbon. For example, clayey soils have on average slower decomposition rates and higher retention of OM than sandy soils and a negative correlation between clay content and decomposition of crop residues is often found [19]. McKenzie and Mason [6], similarly stated that clay soil types in general result in a slower rate of decomposition compared to sandy soils.

There are several below-ground factors that moderate OM decomposition. For example soil moisture content is essential for decomposition, although excessive moisture can lead to anaerobic conditions and reduced the rates of OM breakdown because of a lack of oxygen for soil organisms compared to soils exposed to the atmosphere [6]. de Wit, Lesschen [21], showed that reduced tillage can also promote SOC sequestration by limiting soil disturbance, which reduces decomposition by aeration. However, Chendev, Novykh [22], indicated that accelerated OM decomposition due to coarse textured soils in warm temperatures and low water holding capacity can limit plant growth which can result in low SOC. Scheffer and Aerts [23], stated that roots and rhizomes can play a major role in cycling of carbon and nutrients. But, Amougou, Bertrand [24] indicated that abscised leaves (in their case, of *Miscanthus*) can contribute more to the soil carbon accumulation than rhizomes or roots. Beuch, Boelcke [25], similarly mentioned that *Miscanthus* roots, compared to shoots, have less readily decomposable soluble compounds. Hence, moisture, reduced tillage, soil texture, temperature and below-ground biomass are the factors playing key roles in the SOC sequestration and decomposition. Studies on the decomposition of belowground plant parts are therefore important to fully understand carbon cycling.

In this study, we examined vetiver’s (*Chrysopogon zizanioides*) above- and below-ground biomass production where plants were grown under glasshouse conditions in sandy soil, in addition to the relative decomposition rates of the grass shoot and root biomass when incubated with three Australian soil textures (sand, silt and clay) with different initial properties (e.g., texture, pH and SOC). The aim of this study was to: Quantify the above- and below-ground biomass of vetiver grown in sandy soil under glasshouse conditions; Measure the relative difference in the rate of decomposition of the above- and below-ground biomass of vetiver grass and determine the effect of contrasting soil types on root and shoot decomposition.

## 2. Results

### 2.1. Vetiver Biomass Production

During the seven-month growing period, vetiver produced a mean total of 2.68 kg m^−3^ fresh and 1.2 kg m^−3^ dry biomass (root plus shoot) (Table 1). The above- and below-ground biomass was (1.61 ± 0.218 kg m^−3^) and (1.07 ± 0.128 kg m^−3^) fresh and (0.67 ± 0.101 kg m^−3^) and (0.53 ± 0.054 kg m^−3^) dry biomass, respectively. This translated to a shoot to root biomass ratio of (1:1.43) for the fresh biomass and (1:1.25) for the dry biomass (Table 1). On a per hectare basis, if planted at densities equal to the pot area, the mean total biomass for shoots would equate to 161 Mg ha^−1^, and the root biomass 107 Mg ha^−1^ fresh biomass and 67.7 Mg ha^−1^ dry shoot and 52.5 Mg ha^−1^ dry root biomass. The root biomass decreased exponentially with depth in the pot (Table 2). Mean shoot length was 1.54 m and the roots penetrated to 0.86 m in the 1.0 m pot depth, and the average number of tillers produced was 14 per each planted tiller for the growing period (213 days).

### 2.2. Vetiver Root and Shoot Relative Decomposition Rate

**Figure 1:**
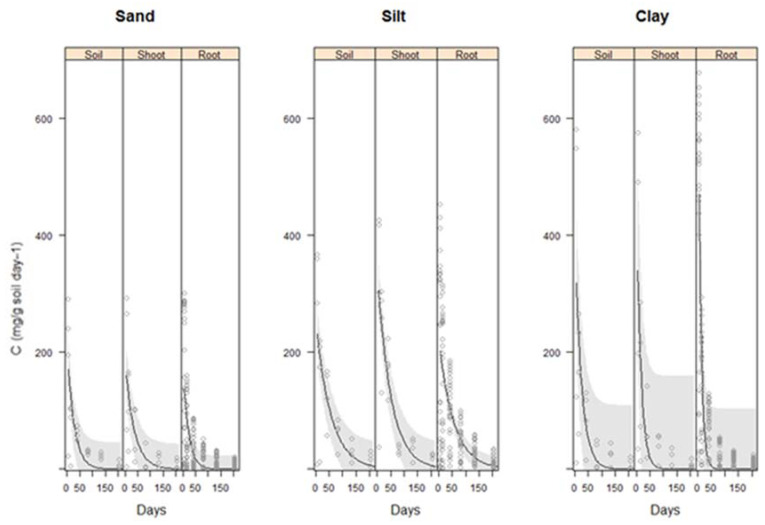
Carbon (mg C g soil^−1^ day^−1^) evolved from vetiver shoot and root decomposition in the three soils during 206−days incubation period. Vetiver biomass was used from the glasshouse experiment and incubated at 25 °C constant temperature. Plots show the raw data (°), a fitted exponential model (–) and 95% confidence bands.

Analysis of variance showed a significant difference in the rate of decomposition of vetiver root and shoot biomass in all soil types (Figure 1 and Table 3). The difference between shoot and root decomposition rate was indicated by a higher rate of decomposition for vetiver roots compared with the shoot biomass in the clay soil type (*p* < 0.001). The difference between shoot and root decomposition was not consistent through the decomposition period for the clay soil. Both root and shoot biomass decomposition differed between soil types where decomposition was higher in the clay soil type compared with the sand and silt soils. For the soils only treatment without vetiver biomass addition, carbon evolved was significantly different depending on time (*p* < 0.001), and this was most pronounced during the first seven days. Carbon evolved from the clay soil was also higher for the first seven days due to the difference in the initial organic matter content between the soils.

### 2.3. Effect of Soil Type on Carbon Evolved from the Vetiver Biomass

**Figure 2:**
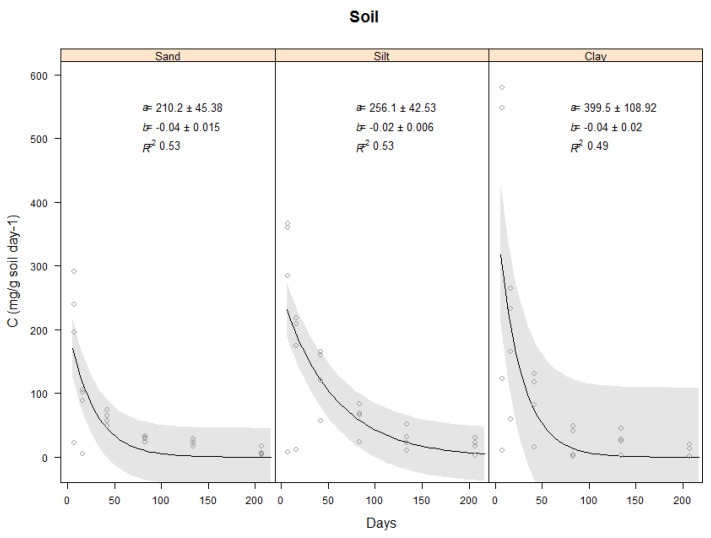
Carbon (mg C g soil^−1^ day^−1^) evolved from the soils (sand, silt, clay) without biomass addition during 206-days decomposition period. Where soils only incubated at 25 °C constant temperature. Plots show the raw data (o), a fitted exponential model (−) and 95% confidence bands. Values of the intercept (a), the slope (b) and Coefficient of determination (R^2^).

**Figure 3:**
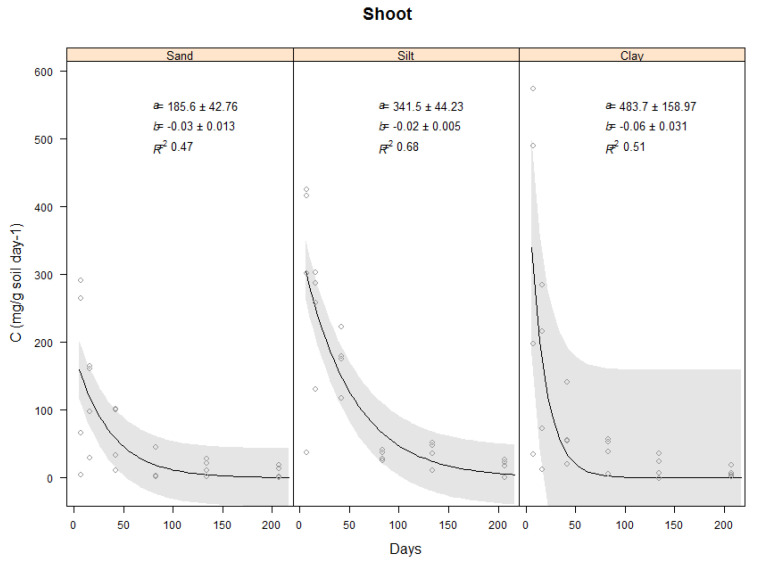
Carbon (mg C g soil^−1^ day^−1^) evolved from the decomposition of vetiver shoot biomass in three soils for 206 days decomposition period. Where vetiver shoot used from the glasshouse experiment and incubated at 25 °C constant temperature. Plots show the raw data (o), a fitted exponential model (–) and 95% confidence bands. Values of the intercept (a), the slope (b) and Coefficient of determination (R^2^).

**Figure 4:**
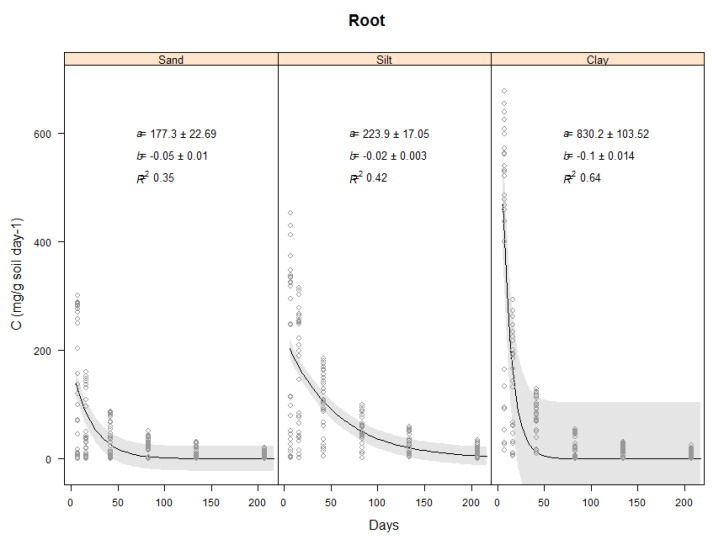
Carbon (mg C g soil^−1^ day^−1^) evolved from blank soils and from the decomposition of the whole vetiver root biomass in three soils during 206 days-incubation period. Where vetiver root biomass used from the glasshouse experiment and incubated at 25 °C constant temperature. Plots show the raw data (o), a fitted exponential model (–) and 95% confidence bands. Values of the intercept (a), the slope (b) and Coefficient of determination (R^2^).

This section will describe the results found for the carbon evolved from the controls which were the three soil types (Sand, Silt and clay) only (Figure 2) and the carbon evolved from the vetiver shoots and roots in the three soil types (Figure 3 and Figure 4).

#### 2.3.1. Carbon Evolved from Three Soil Types

For the sand, silt and clay soils without vetiver addition, carbon evolved followed a double exponential decay curve where it began with rapid phase and then slow phase (*p* < 0.001), and this was most pronounced during the first seven days. Carbon evolved from the clay soil was the highest during the first seven days compared with the silt and the sand. The analysis indicated that the carbon evolved from the clay soil type was significantly higher compared with silt and sand soil types (*p* = 0.001). For all three soil types, C evolution began to plateau after Day 43 (Figure 2).

#### 2.3.2. Effect of Soil Type on Carbon Evolved from Vetiver Shoots

Analysis of variance indicated that the shoot decomposition rate was affected by soil type. CO_2_ evolved from the clay soil was the highest at 323.9 mg C day^−1^ for the first 7 days, compared to 294.9 mg C day^−1^ for the silt and 156.5 mg C day^−1^ for the sand. The analysis indicated that the difference between the shoot decomposition in the clay and silt soils were significantly different (*p* = 0.001), and that both were significantly higher than in the sand (*p* = 0.001). The total carbon evolved from shoot decomposition in the clay soil was the most rapid, particularly during the first seven days, and as with the soil only, slowed only after Day 43 (Figure 3).

#### 2.3.3. Effect of Soil Type on Total Carbon Evolved from Vetiver Roots

Analysis of variance indicated that the amount of carbon evolved from vetiver root decomposition, for all depths combined, was affected by the soil type and time (*p* < 0.001). However, the differences between soil types were only in the early stages of the experiment. For soil type, carbon was evolved from vetiver root decomposition in the clay soil (average for first 7 days 413.18 mg C day^−1^) was higher compared to the silt (average for first 7 days 205.37 mg C day^−1^) and sandy (average for first 7 days 142.34 mg C day^−1^) soils (*p* < 0.001) (Figure 4).

**Figure 5:**
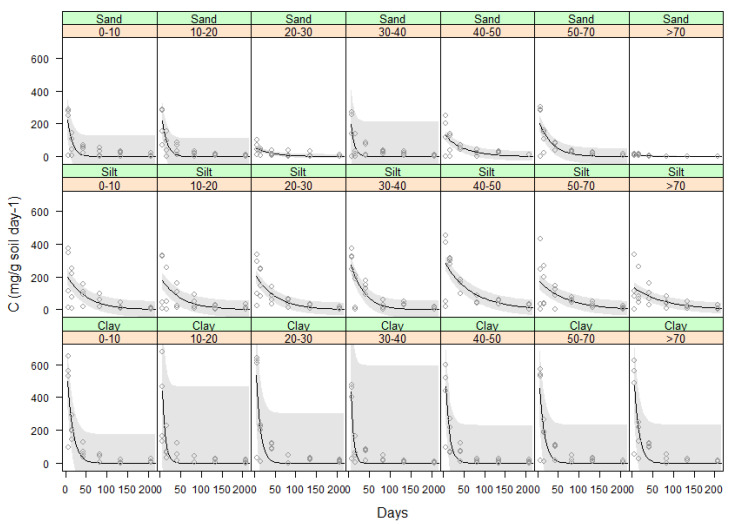
Carbon (mg C g soil^−1^ day^−1^) evolved from the decomposition of vetiver root biomass of seven depth increments in three soils during 206 days. Where vetiver root biomass used from the glasshouse experiment and incubated at 25 °C constant temperature. Plots show the raw data (o), a fitted exponential model (–) and 95% confidence bands.

Vetiver root decomposition was also analysed for individual biomass produced at different soil depth taken through the 1.0 m pots which were divided into 7 depth increments (0–0.1, 0.1–0.2, 0.2–0.3, 0.3–0.4, 0.4–0.5, 0.5–0.7 & 0.7–1.0 m). Therefore, vetiver root decomposed at the same rate regardless of where they are sampled (depth) and was consistent (Figure 5). Hence, there was no significant difference between decomposition in the clay and silt soils. The proportion of the variance in the carbon evolved predicted by time was above *R*^2^ = 0.6 for the clay and lower for the silt and sand (Table 4).

## 3. Discussion

### 3.1. Vetiver Biomass Production

Many studies have shown that vetiver has the potential to produce a large amount of biomass both above- and below-ground and have therefore suggested it has the potential to store additional carbon [26,27,28,29,30]. Hence, we recorded more vetiver above- and below-ground dry biomass production potential across a seven-month growing period (67.7 Mg ha^−1^ and 52.5 Mg ha^−1^, respectively) compared with all previous reported data. Tomar and Minhas [30], who examined different cultivars of vetiver in India for two years growing period, found 72.6 to 78.7 Mg ha^−1^ shoot and 1.12 to 1.71 Mg ha^−1^ root biomass, and a total mean dry biomass for vetiver 30.3 Mg ha^−1^ year^−1^. In contrast, a study by Neal, Fulkerson [31] indicated the dry biomass production potential of vetiver as only half (17 Mg ha^−1^ year^−1^), of the value reported by Tomar and Minhas [30]. Vetiver has been reported to produce28.62 Mg ha^−1^ year^−1^ dry shoot and 1.56 Mg ha^−1^ year^−1^ dry root biomass in Thailand and India, respectively [27,32]. For *Andropogon guayanus*, a similar tropical perennial grass a 43 Mg ha^−1^ year^−1^ of above ground biomass in the South American Savannah [9] was reported. For another similar grass, *Miscanthus*, Amougou, Bertrand [24], indicated 18.5 to 21 Mg ha^−1^ year^−1^ dry biomass production potential in Northern France which on average is equivalent to vetiver dry biomass (17 Mg ha^−1^ year^−1^).

In this study, we also found an average of 14 tillers per plant which is higher than previously reported results. For example Xu [33], reported only 4–6 tillers per vetiver plant in the northern subtropics of China and Kaveeta, Sopa [32], reported 7–8 tillers/plant for four ecotypes of vetiver grown in China which is on average half of the result under this study.

The variation in biomass production reported in the literature is probably a consequence of the specific growing conditions (i.e., optimum moisture, nutrient supply and temperature) [26], and variations in the genetic potential of the germplasm used [29]. Also, the planting density used to calculate biomass production does not consider any spacing. If biomass production in our trial was applied as a standard vetiver planting rate of 88.5 plants ha^−1^ [34], the potential production would be 120 Mg ha^−1^, which is still significantly higher than the values reported by other workers above.

These conditions did not occur in some other studies [26,35,36] which may explain why shoot and root biomass levels in this study were much higher than those reported for vetiver grown in field conditions. Our result indicates that vetiver with good agronomic practices during establishment (i.e., nutrient addition, regular watering) has the potential to produce a large amount of biomass when it is planted in carbon and nutrient depleted soils. Hence, the data obtained are useful for parameterization of ecosystem services for depositing carbon in soils since the data is found under ideal model greenhouse conditions.

### 3.2. Vetiver Biomass Decomposition

Vetiver roots decomposed more rapidly than the shoots in the clay soil. This is due in part to the lower C:N ratio of the roots compared with the higher C:N ratio of the shoot biomass (1:21 and 1:66, respectively). A comparative study between buried and mulched vetiver shoots by Chairoj and Roongtanakiat [34], demonstrated that the rate of decomposition was higher in buried shoots compared to mulched vetiver shoots, and this can be due to more contact between the plant biomass and soil. Soil moisture, temperature, organic carbon concentration and microbial activity are all affected by incorporation of plant residues in soils, all of which can further influence decomposition of plant material [37]. In our study, the biomass was placed on the soil surface where the root and shoot material had less soil contact, and the comparison was between the above and below ground biomass and a faster decomposability of the vetiver root was observed compared with the shoot biomass. So, it might be expected that incorporation of root or shoot material would increase decomposition rate. The faster decomposition of vetiver root can potentially explain the exponential decrease in soil carbon with increasing soil depth and a rapid decomposition rate of vetiver in the soil may result in high carbon turnover [20,38,39,40,41,42,43,44]. Hence, the higher decomposition of vetiver root exudates is crucial because it releases the carbon in the root exudates and would also speed up its contribution to stable soil organic matter. On the other hand, the slowness of vetiver shoot decomposition could hold the C in decomposing litter longer but would also slow down its contribution to stable soil organic matter this is in agreement with [45].

A C:N ratio of 1:30 is commonly regarded as a threshold for predicting whether net N mineralization (<30) or net N immobilization (>30) occurs following crop residue addition, although this empirical parameter can vary from one soil to another [46]. Plant materials of different C:N ratios affect bacterial and fungal growth differently, leading to further variations in the C:N ratio of newly produced microbial biomass [47,48]. Irrespective of soil aeration, Li, Hu [42], reported that soil N_2_O production was generally lower using plant materials with high C:N ratios compared to those with low C:N ratios. The C:N ratio of vetiver is generally reported as 19–79 [49], and in this study it was 66 for shoot and 21 for roots. This suggests that N mineralization was likely for the root material while immobilisation of N was more likely to occur for shoot material, with related effects on the rate of decomposition and the storage of carbon in the soil.

Carbon evolved from both shoot and root biomass in the three different soil types indicated that soil type can affect the rate at which above- and below-ground vetiver biomass decomposes. CO_2_ evolution was greater in clay soils and decomposition was faster than in the sand and silt soils for both shoot and root biomass. This effect was more pronounced in the first 50 days, before it started levelling out for all three soil types for the next 150+ days, similar to other findings [50,51,52]. Although a study by Bronick and Lal [18] showed that clay content can suppress decomposition and promote SOC storage by increasing the physical protection of SOC within soil aggregates, in our study the biomass was placed on the soil surface, minimising the potential protective effects of the soil aggregates in the clay and the physicochemical processes (adsorption) occurring on the clay particles. Instead, the faster decomposition in the clay soil compared with the silt and sand was more likely due to different initial nutrient and moisture levels, pH, structure, biological activity and a combination of these [14,15,16].

Roots decomposed faster than shoots but this was not consistent through the whole experiment period which could be an initial flush of labile C (with lower C:N) followed by a slowing and convergence of decomposition of shoot and root behavior. The implications of the results from study are, therefore, roots decompose faster compared to shoots however, they decompose at the same rate regardless of where they are sampled (depth) from.

## 4. Materials and Methods

### 4.1. Experimental Setting and Design

To determine the above- and below-ground biomass production and relative decomposition of vetiver (*Chrysopogon zizanioides*) grass, an experiment was undertaken from late 2014 through to 2016 at the University of New England, Australia. Specimens of vetiver were collected in March 2014 from the NSW Office of Environment and Heritage, Gunnedah Research Centre, NSW where the vetiver had been established for more than 20 years. Specimens were vegetatively propagated and then maintained in a UNE glasshouse until required. Prior to the experiment, a three-month pilot experiment was conducted in a glasshouse where vetiver was planted in 12 small pots (0.3 m height × 0.12 m diameter) with a sand textured soil. Within three months, vetiver roots had reached the bottom of the pots, therefore, we estimated that a six-month growth period would be suitable for vetiver root extension in 1.0 m length pots for the subsequent experiment.

### 4.2. Treatment Description

A sandy loam soil was used for biomass production so that the root biomass could be recovered easily. The soil was collected from 0–20 cm (top) soil depth from the UNE Newholme Farm and had a sandy loam texture and extremely low fertility (see Table 5. for soil characterization). We added 4 g of multi-grow fertilizer (10.1% N, 3.5% P, 5.5% K, 16.3% S and 7.8% Ca) via surface fertilizer application to pots at the same point as root cuttings (~6 cm depth) to provide starter nutrients to the 10 replicate pots (radius = 6 cm, height = 1.0 m, area = 113.04 cm^2^). The resultant biomass from five randomly selected pots was used to measure shoot and root biomass production, and fresh biomass (refrigerated) from two randomly selected pots supplied plant material for the decomposition experiment (Table 6). For each pot, a single vetiver plant was split and cut to a 0.06 m root and 0.12 m shoot length, then planted and watered to up to 60% Field Capacity [53]—follow-up watering to the same moisture content was conducted every second day of the experiment under ideal ambient temperature and humidity glasshouse condition.

### 4.3. Sample Collection, Preparation & Analysis

#### 4.3.1. Biomass Production

The extent of root growth and its extension through the entire soil volume in selected pots was determined by CT-scanning during month six of the experiment to re-confirm a sufficient root mass had established in the pot and plants were suitable for harvesting—any further extension may have been restricted by the pot. At the end of the experimental period (213 days), five of the 10 pots were randomly selected, and vetiver harvested for above- and below- ground biomass assessment while the other five were used for the decomposition experiment. Soils from each pot were divided into the following seven depth increments to vertically differentiate the root biomass: 0–0.1 m, 0.1–0.2 m, 0.2–0.3 m, 0.3–0.4 m, 0.4–0.5 m, 0.5–0.7 m, and >0.7 m and the whole root mass extracted from each by washing with distilled water. Fresh shoots and roots were weighed to provide fresh biomass, then dried at 70 °C to provide dry biomass. A total of 5 shoot biomass samples and a total of 35 root biomass samples (5 × 7 depth increments) were used for biomass production analysis. Shoot to root ratio (g dry matter^−1^ m^−2^ of each depth increment), shoot/root length (m) and number of stems were counted and measured.

#### 4.3.2. Decomposition

The decomposition study was conducted in 2016 for 206 days to assess vetiver shoot and root biomass decomposition when applied to three different soils: Sandy-Chromosol (Kirby), Silty-Chromosol (Dalkieth) and Clayey-Dermosol (Clarke’s farm) [54,55]. The soil types will be hereafter referred to as Sand, Silt and Clay; respectively. The three soils had different initial properties (Table 5). The field capacity was calculated as 0.7027, 0.3576 and 0.3377 g water g^−1^ soil for the clay, sand and silt soils, respectively. We used sealed polypropylene jars (250 mL) with lids fitted with septa to facilitate gas sampling during incubation of soil and plants material. The fresh biomass was supplied from two pots randomly selected from the 10 replicate pots established during the biomass assessment, and resultant shoot and root (divided into the seven depth increments detailed above) biomass were refrigerated until the incubation installation. Fresh vetiver shoot and root biomass (properties detailed on Table 7) samples were chopped to between 5–10 mm and then added to each soil surface in each jar. Both the shoot and root to soil ratio was 1:500 that is 0.05 g of shoot or root materials in 25 g soil, with four replicates of each soil to biomass mixture. The treatments used in this experiment therefore are soils (3 types) × shoot (1 level) × root (7 levels) = 21. A blank was set for the purpose of calculating actual emission by considering the blank as an ambient concentration. Total jars = 21 treatments × 4 replicates = 84 jars excluding the blanks. Four replicate controls of each soil type with no biomass material added were also included, along with four blank containers (*n* = 112) (Table 8). Containers were placed in a constant temperature (25 °C) cabinet in the dark. Six gas samples were taken from each vessel during the incubation period (at Day 7, 16, 42, 83, 134 and 206). The sampling dates were randomly taken considering the decaying trend for decomposition and samples were taken in the beginnings of the dates mentioned. At each sampling time, 12mL vacutainers were evacuated and a gas sample extracted from the headspace within each container. Following this, jars were opened to the ambient air and watered to achieve 60% field capacity. Septa were then replaced and jars re-sealed and returned to the constant temperature environment until the next sampling time.

CO_2_ evolved per day was evacuated for each measurement period following Equation (3), and this value was calculated as an average CO_2_ evolved per day over each sampling period. An ANCA-GSL combined elemental analyser and gas purification module that produces clean gas samples for a 20–20 isotope ratio mass spectrometer was used for analysis of the gases. The experiment measured CO_2_ evolved from soil only and soil plus fresh (shoot and root) biomass through time for four replicate samples: soil only (sand, silt, clay); shoot × soil (sand, silt, clay) and root × soil (sand, silt, clay), with values from blank containers subtracted from treatment values (Table 8).

### 4.4. Data Analysis

For shoot and root biomass production we calculated the mean for the five replicate shoot samples and five replicate root samples for each of the seven depth increments. Root and shoot biomass are reported as mass per unit volume (mv^−1^) of soil in the soil pots [pot surface area × pot height (1.0 m)] in kg m^−3^ accumulated during the 7-month growth period. Where, the diameter of the pot was 0.12 m.

For the decomposition experiment, CO_2_ evolved was calculated by deducting the blank from the measured samples. Adjustments were made during gas chromatography [38,56,57,58] measurement for container volume, soil mass and number of days of incubation Equation (1). Carbon dioxide evolved per gram of soil was calculated following Equation (2) and results presented in mg C g^−1^ soil day^−1^, where the CO_2_ evolved was converted to C [20,34,41,42,57,59] using Equation (3).

(1) $${\mathrm{CO}}_{2}\left(\mathrm{released}\right)=\frac{\left[{\mathrm{CO}}_{2}\left(\mathrm{measured}\right)-{\mathrm{CO}}_{2}\left(\mathrm{blank}\;\mathrm{container}\right)\right]}{\mathrm{Duration}\left(\mathrm{Days}\right)}$$

(2) $${\mathrm{CO}}_{2}=\frac{{\mathrm{CO}}_{2}\left(\mathrm{released}\right)}{g\;\mathrm{Soil}}$$

(3) $$C\left(\mathrm{mg}\right)=3.67{\mathrm{CO}}_{2}$$

The statistical analyses were performed using R version 3.3.2. One-way analysis of variance (ANOVA) was performed to test if there was an effect of time on decomposition and to determine differences between different soil types. Tukey’s honestly significantly different (HSD) test was then performed to determine statistically significant differences (*p* < 0.05). Non-linear regression was used to analyse the single no response variable (the carbon evolved) over time. An exponential decay function Equation (4) was used,

(4) $$y=a\times \exp^{\left(-b\right)}$$

where *y* = carbon, *a* = y-intercept and *b* = decay constant (>0).

## 5. Conclusions and Implications

The results from this study confirmed the large biomass (both above- and below-ground) production potential of vetiver grass over a short period of time even in soils with low fertility. The application of vetiver shoots and roots biomass on the surface of three soils with contrasting textures has also decomposed differently over time and the decomposition was more rapid in the clay soil compared with the sand and silt soils. However, the rate of decomposition of vetiver roots was more rapid than the shoots in all soil types. Besides, the high biomass production potential, the more rapid decomposition rate of vetiver root materials regardless of where they were sampled (root depth) from could be attributable to the lower C:N ratio of the vetiver roots compared with the vetiver shoots. Hence, the larger carbon storage through the depth and deeper soils could be a contribution from the vetiver roots than the shoots. This research, therefore, suggests that the large root biomass of vetiver contributed more to the soil carbon accumulation not only to the soil organic matter than the shoot biomass. This is due to the faster decomposition of vetiver root exudates which is crucial in releasing the carbon in the root exudates and would also speed up its contribution to stable soil organic matter. Hence, planting vetiver and similar tropical perennial grasses on degraded and less fertile soils could be a good strategy for carbon sequestration and to rehabilitate degraded soils. We, therefore, suggest that farmers need to be encouraged to plant vetiver and similar tropical perennial grasses on degraded soils and marginal lands to facilitate rehabilitation and carbon sequestration. Further research also needs to be conducted to investigate the mechanisms and impacts of potential tropical grasses like vetiver.

## Figures and Tables

**Table 1:** Mean and ratio of vetiver dry and fresh biomass (shoot to root) production (kg m^−3^) plantation in 1.0 m pot in a sand soil for the experimental period (7 month).

Plant Allocation	Fresh Biomass (kg m^−3^)	Dry Biomass (kg m^−3^)
Above-ground biomass	1.61 ± 0.218	0.67 ± 0.101
Below-ground biomass	1.07 ± 0.128	0.53 ± 0.054
Total biomass	2.68 ± 0.344	1.2 ± 0.151
Shoot−to−Root ratio	1:1.43	1:1.25

**Table 2:** Mean fresh and dry root biomass production for the seven root depth increments of a 1.0 m pot.

Root Depth (cm)	Fresh (g)	Dry (g)
0–10	56.2 ± 29.9	28.9 ± 13.5
10–20	17.7 ± 7.1	9.4 ± 1.7
20–30	14.6 ± 6.6	7.1 ± 2.8
30–40	12.5 ± 6.0	5.3 ± 2.7
40–50	8.4 ± 4	3.6 ± 1.8
50–70	9.3 ± 7.8	3.7 ± 3.2
>70	2.8 ± 5.4	1.6 ± 3.1

**Table 3:** Rate of vetiver biomass (above and below ground biomass) decomposition (mg C g^−1^ soil) in three soil textures (sand, silt and clay). Values of the intercept (a), the slope (b) and Coefficient of determination (R^2^).

	a			b			R^2^		
	Sand	Silt	Clay	Sand	Silt	Clay	Sand	Silt	Clay
Soil	210 ± 45	256 ± 43	400 ± 109	−0.04 ± 0.015	−0.02 ± 0.006	−0.04 ± 0.02	0.53	0.53	0.49
Shoot	186 ± 43	342 ± 44	484 ± 159	−0.03 ± 0.013	−0.02 ± 0.005	−0.06 ± 0.031	0.47	0.68	0.51
Root	177 ± 23	224 ± 17	830 ± 104	−0.05 ± 0.01	–0.02 ± 0.003	−0.1 ± 0.014	0.35	0.42	0.64

**Table 4:** Rate of carbon evolvement from vetiver below ground biomass decomposition (mg C g soil^−1^ day^−1^) in three soils. Values of the intercept (a), the slope (b) and Coefficient of determination (R^2^).

Root Depth (cm)	a			b			R^2^		
	Sand	Silt	Clay	Sand	Silt	Clay	Sand	Silt	Clay
0–10	364 ± 125	225 ± 48.5	756 ± 173	−0.090 ± 0.04	−0.021 ± 0.01	−0.074 ± 0.02	0.55	0.44	0.74
10–20	355 ± 110	198 ± 50.5	967 ± 466	−0.090 ± 0.03	−0.020 ± 0.01	−0.141 ± 0.06	0.62	0.35	0.6
20–30	58 ± 12.7	226 ± 38.8	919 ± 301	−0.020 ± 0.01	−0.020 ± 0.01	−0.097 ± 0.04	0.42	0.54	0.64
30–40	399 ± 212	324 ± 53.2	1130 ± 595	−0.130 ± 0.06	−0.031 ± 0.01	−0.172 ± 0.07	0.42	0.65	0.64
40–50	142.0 ± 3	305 ± 46.7	791 ± 230	−0.021 ± 0.01	−0.015 ± 0.01	−0.095 ± 0.03	0.41	0.54	0.7
50–70	242 ± 49	184 ± 44.3	734 ± 232	−0.032 ± 0.01	−0.015 ± 0.01	−0.085 ± 0.03	0.55	0.31	0.62
70–100	115 ± 2.6	144 ± 33.0	764 ± 234	−0.033 ± 0.01	−0.013 ± 0.01	−0.086 ± 0.03	0.65	0.32	0.63
0–100	177 ± 23	224 ± 17.0	830 ± 104	−0.500 ± 0.01	−0.02 ± 0.003	−0.1 ± 0.014	0.35	0.42	0.64

**Table 5:** Physical and chemical characteristics of the soils used for the biomass production and decomposition experiments.

Soil Type	Texture	Sand %	Silt %	Clay %	SOC %	δ^13^C %	% TN	δ^15^N	C:N	pH
Sandy-Chromosol	Sandy loam	76.30	10.50	13.20	1.06	−18.39	0.09	0.37	0.09	4.4
Silty-Chromosol	Silty clay loam	45.00	25.00	30.00	2.25	−19.80	0.20	0.37	0.44	5.2
Clayey-Dermosol	Silty clay	19.00	26.00	55.00	2.03	−19.23	0.14	0.37	0.29	6.4

**Table 6:** Biomass assessment design used for biomass and decomposition experiment.

Experiment	No. of Pots	Reps	Biomass	Materials Used	Activity	Analysis
Biomass production	5	5	Fresh	Shoot and Root	Harvesting	Measure and weighing
		5 ^1^	Dry	Shoot and Root		
Decomposition ^2^	2	4	Fresh	Shoot	Incubation	GC-MS
		4 × 7 depth	Fresh	Roots		
		4	None	Soils		

^1^ The 5 dried reps were the 5 fresh reps dried after fresh mass was recorded. ^2^ Fresh plant supply only for further decomposition study.

**Table 7:** Properties of above- and below-ground biomass of vetiver grass.

Vetiver	C (%)	δ^13^C	N (%)	C:N
Shoot	44.10	−11.59	1.49	66
Root	34.22	−13.87	0.61	21

**Table 8:** Decomposition experiment design including blanks and the three soil types with no biomass added for reference, and soils (sand, silt and clay) with shoot and root (seven depth increments) biomass added.

	Sand	Silt	Clay	Soil Type	Rep	Depth	Total Sample
Blank	-	-	-	-	4	-	4
Soil	Sand	Silt	Clay	3	4	-	12
Shoot	Shoot + Sand	Shoot + Silt	Shoot + Clay	3	4	-	12
Root	(Root * + Sand)	Root * + Silt	Root * + Clay	3	4	7	84
	root _(0–10 cm)_ + sand	root _(0–10 cm)_ + silt	root _(0–10 cm)_ + clay				
	root _(10–20 cm)_ + sand	root _(10–20 cm)_ + silt	root _(10–20 cm)_ + clay				
	root _(20–30 cm)_ + sand	root _(20–30 cm)_ + silt	root _(20–30 cm)_ + clay				
	root _(30–40 cm)_ + sand	root _(30–40 cm)_ + silt	root _(30–40 cm)_ + clay				
	root _(40–50 cm)_ + sand	root _(40–50 cm)_ + silt	root _(40–50 cm)_ + clay				
	root _(50–70 cm)_ + sand	root _(50–70 cm)_ + silt	root _(50–70 cm)_ + clay				
	root _(>70 cm)_ + sand	root _(>70 cm)_ + silt	root _(>70 cm)_ + clay				
		Total no. of samples					112

* Root at a specific soil depth.

## Data Availability

Not applicable.
